# Case report: A Chinese patient with spinocerebellar ataxia finally confirmed as Gerstmann-Sträussler-Scheinker syndrome with P102L mutation

**DOI:** 10.3389/fneur.2023.1187813

**Published:** 2023-08-03

**Authors:** Lin Chen, Yin Xu, Ming-juan Fang, Yong-guang Shi, Jie Zhang, Liang-liang Zhang, Yu Wang, Yong-zhu Han, Ji-yuan Hu, Ren-min Yang, Xu-en Yu

**Affiliations:** Department of Neurology, The Affiliated Hospital of Institute of Neurology, Anhui University of Chinese Medicine, Hefei, China

**Keywords:** Gerstmann-Sträussler-Scheinker syndrome, *PRNP* gene, P102L, spinocerebellar ataxia (SCA), prion disease

## Abstract

Gerstmann-Sträussler-Scheinker syndrome (GSS) is a rare genetic prion disease caused by a mutation in the prion protein (*PRNP*) gene. It is typically characterized by progressive cerebellar ataxia and slowly progressive dementia. We present a case study of the GSS from China in which a 45-year-old male with a progressive gait and balance disorder developed cerebellar ataxia onset but was misdiagnosed as spinocerebellar ataxia (SCA) for 2 years. The patient's clinical, electrophysiological, and radiological data were retrospectively analyzed. Examination revealed ataxia, dysarthria, muscle weakness, areflexia in lower limbs, including a pyramidal sign, whereas cognitive decline was insignificant. His late mother had a similar unsteady gait. An electroencephalogram (EEG) showed normal findings, and 14-3-3 protein was negative. A brain MRI was performed for global brain atrophy and ventricular enlargement. Positron emission tomography–computed tomography (PET–CT) (18F-fluoro-2-deoxy-d-glucose, FDG) images showed mild to moderate decreased glucose metabolism in the left superior parietal lobe and left middle temporal lobe. According to genetic testing, his younger brother also had the P102L variant in the *PRNP* gene. This single case adds to the clinical and genetic phenotypes of GSS.

## Background

Gerstmann-Sträussler-Scheinker syndrome (GSS) is a rare genetic fatal prion disease with clinical heterogeneity where the prevalence ranges from 1 to 10 per 100 million individuals and is characterized by progressive cerebellar dysfunction and cognitive decline (1). GSS was initially described as a rare familial disease of the central nervous system. In 1995, a proline-to-leucine mutation at codon 102 (P102L) in the *PRNP* gene was identified in a family (2). Although the P102L mutation has been reported in several Chinese GSS cases, it may not be a common mutation in China (3). GSS syndrome with P102L mutation was first reported in China in 2006, and only 20 cases with P102L-associated GSS have been reported so far (Table 1) (7, 8, 12, 13, 15, 17, 18, 21, 23).

**Table 1:** Comparison of basic features of GSS cases with P102L mutation previously reported in Asian region.

**References**	**Patient no**.	**Origin**	**Sex/age at onset**	**Clinical symptoms**	**Genotype**	**14-3-3**	**EEG**	**Neuroimaging**	**Neuropathology**
Tanaka et al. (4)	1–6	Japanese	6 cases; 1 family 2 in detail 34 y/m 64 y/m	Mental deterioration, speaking and writing difficulty, reduction in verbal fluency, mild ataxia, and wide based gait	P102L–E219K	N	Normal	Atrophy in the cerebral cortex, multiple ischemic lesions	No spongiform changes, no neuronal loss, mild to moderate gliosis, diffuse cortical plaques
Yamada et al. (5)	7–8	Japanese	2 cases; 39 y/f, 35 y/f	Dysarthria, ataxic gait, dysesthesia painful paresthesias, speech disturbances, dysarthria, nystagmus, areflexia in the legs	P102L−129M/M	N	Frequent bursts of theta waves in frontal leads without periodic synchronous discharges	Slight cerebellar atrophy (Case 1)	Spongiform changes in the cerebral and cerebellar cortices, kuru-type plaques, PrP deposits in brain and spina
Arata et al. (6)	9–19	Japanese	11cases; 38–70 y (nine families)	Gait disturbance; dysesthesia; hyporeflexa of lower legs; truncal ataxia; leg muscle weakness; dementia and mutism	P102L–M129	Positive in two patients	Normal	High-intensity cerebral cortex, lesions in occipital lobes; others: atrophy	N
Wang et al. (7)	20	Chinese	33 y/F	Dementia and cerebellar ataxia rapidly progressing; language and cognition became progressively more disturbed	P02L	N	Paroxysmal slow waves without periodic synchronous discharges	The upper thoracic segments and mild cerebellar atrophy	Moderate spongiform changes and neuronal loss in the cerebral cortices; proliferation of hypertrophic astrocytes in the cerebral cortices diffuse amyloid plaques in the cerebral cortices; amyloid plaques showed strong immunopositivity by anti-PrP;
Chi et al. (8)	21–27	Taiwan	7 cases; 37–53 y	Difficulty to walk, leg weakness, unsteadiness, dysarthria, depression	P102L–M129	N	1 case: diffuse slow activity; others: normal	3 cases: mild cerebellar atrophy, others: normal	N
Min Jeong Park et al. (9)	28	Korea	1 case; 46 y/f	Slowly progressive ataxia; cognitive decline; dysarthria; severe dementia; dyskinesias	P102L	Positive	Non-specific generalized theta–delta slow waves	Hyperintensities over the entire hemispheric cortices	N
Takazawa et al. (10)	29	Japanese	1 case; 38 y/f	Dysarthria, agraphia, cerebellar ataxia, insomnia; leg hyperreflexia	P102L–M129	Positive	Diffuse theta and delta waves	Vermis atrophy, fronto-parietal cortical high signal	N
Yasushi Iwasaki et al. (11)	30	Japanese	54 y/f	Dementia and gait disturbance; bedridden state with myoclonus, akinetic mutism state	P102L	N	Diffuse slowing without periodic sharp-wave complexes	Widespread cerebral cortical hyperintensity	Numerous PrP immunopositive plaques and diffuse synaptic-type PrP deposition were extensively observed, particularly in the cerebral and cerebellar cortices
Long et al. (12)	31	Chinese	47 y/f	Unstable gait and dysarthria; speech slurred; dementia, anxiety, depression, hallucinations or delusions	P102L	N	Normal	Cavum vergae, and mild diffuse brain atrophy; intervertebral herniation in C5/6 and C6/7	N
Li et al. (13)	32–36	Chinese	5 cases: 43–55 y	Unsteady walking, dysarthria, dysphagia, changes in personality and irritation, constipation, increased salivation, somnipathy dyssomnia, dementia	P102L	N	Normal	Normal	N
Atsuhiko Sugiyama et al. (14)	37–38	Japanese	2 cases: 55 y/f, 66 y/f	Developed difficulty in using chopsticks, mild speech slurring, subtle dysphagia	P102L	N	N	Atrophy of the cerebellar vermis and brainstem; hyperintensity in the medial portion of both thalami and both pulvinars	N
Wang et al. (15)	39	Chinese	1 case; 49 y/f	Progressive unsteady gait in early stage; progressive dementia; myoclonus; akinetic mutism	P102L	Positive	Dispersedly distributed medium waves together with sharp waves that discharged paroxysmally	Enlarged sulci in cerebellum; high signal intensities in bilateral frontal, parietal, temporal, and occipital cortices	N
Michiyoshi Yoshimura et al. (16)	40–44	Japanese	5 cases; 73 y/f, 62 y/f, 61 y/f, 60 y/m, 59 y/m	Ataxia of lower limbs; gait disturbance; dysesthesia in legs; lower limb hyporeflflexia	P102L	N	Normal	SPECT and PET:blood flow of tnterior cerebellar lobes lower than the posterior cerebellar lobes	N
Wang et al. (17)	45–56	Chinese	12 cases; 34 y-67 y	Movement symptoms (gait and walking instability); mental problems (anxiety, dystrophy, irritability); memory decline, dementia	P102L	Positive from 5 cases (45.5%)	2 (25%) of 8 cases exhibited periodic sharp wave complexes	High signal intensities in caudate/putamen (3 cases), DWI ribbon-like signals (3 cases)	N
Zhao et al. (18)	57	Chinese	48 y/m	Unsteady walking; dysarthria; involuntary head tremors; unbearable muscle pain in both lower limbs	P102L	N	Normal	Normal	N
Min Ju Kang et al. (19)	58	Korea	49 y/m	Progressive gait disturbance, slurred speech, clumsiness in both hands; dysarthria and ataxia	P102L	Positive	Normal	Hyperintensities of bilateral cortices; right anterior putamen, right caudate, mild cerebellar atrophy	N
Kazumichi Ota et al.(20)	59–61	Japanese	1 family: 3 cases; 32 y/m, 53 y/m, 56 y/f	Cognitive function declined; movement symptoms (gait and walking instability), mental problems (behave abnormally); Myoclonus	P102L	Positive	Normal	High signals in occipital and frontal cortices; thalamus and cerebellum mild atrophy	N
Cao et al. (21)	62	Chinese	49 y/m	Unsteady walk with mogilalia; dysdipsia, dysarthria, dizziness, diplopia	P102L	N	N	Cerebral and cerebellar atrophy	N
Yazawa et al. (22)	63	Japanese (Asia)	56 y/f	Worsening dizziness and walking instability; dysarthria	P102L	N	Periodic focal sharp activity in both temporal areas	Mild atrophy of the cerebellum	N

We described a Chinese patient with GSS and a heterozygous mutation in the *PRNP* gene with progressive ataxia, pyramidal signs, and areflexia. The patient had a few cognitive declines previously misdiagnosed as spinocerebellar ataxia (SCA). This case report describes an unusual clinical condition with a positive family history confirmed by gene testing. Our patient and his younger brother both had heterozygous mutations in exon 2 of *PRNP*, located on chromosome 20. A pathogenic mutation causes the P102L mutation at codon 102 in PRNP, the most common variant associated with GSS.

## Case presentation

A 41-year-old Chinese man was referred for an abnormal gait suggestive of ataxia. The patient's physical and intellectual level in early life was normal, but his family noticed decreased language fluency at the age of 40 years. One year later, he was 41-years-old, he often fell due to progressive aggravation of walking instability and decreased muscle strength in his lower limbs. He was treated at hospital at the age of 42 years for ataxia, and he was given buspirone. He deteriorated over time, when he was 44-years-old, he could not walk, and began using a wheelchair. There was no further decline in cognitive status over time.

He had a family history of similar symptoms in his mother. She presented to medical attention at the age of 55 years with an unsteady gait. She required a wheelchair by age 58 years, owing to progressive walking instability and decreased muscle strength in her lower limbs. She was subsequently bedbound but did not attend the hospital for a physical examination and finally died at the age of 60 years. During this time, her family did not realize significant cognitive difficulties. The cause of death was unknown, and her family could not provide further details.

**Figure 1:**
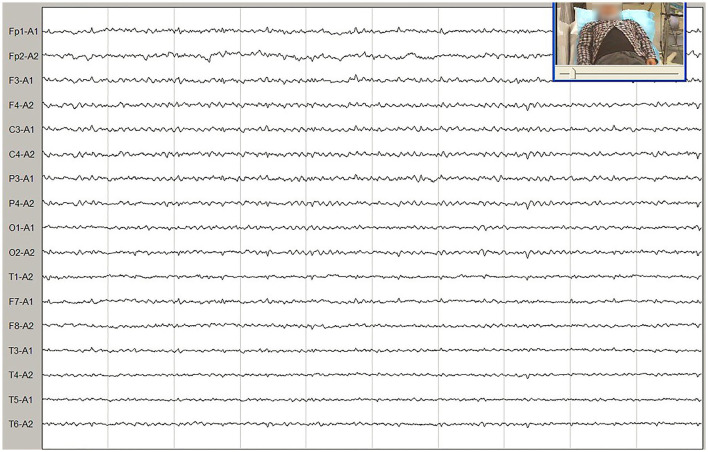
Video electroencephalogram (EEG) showed normal findings.

**Figure 2:**
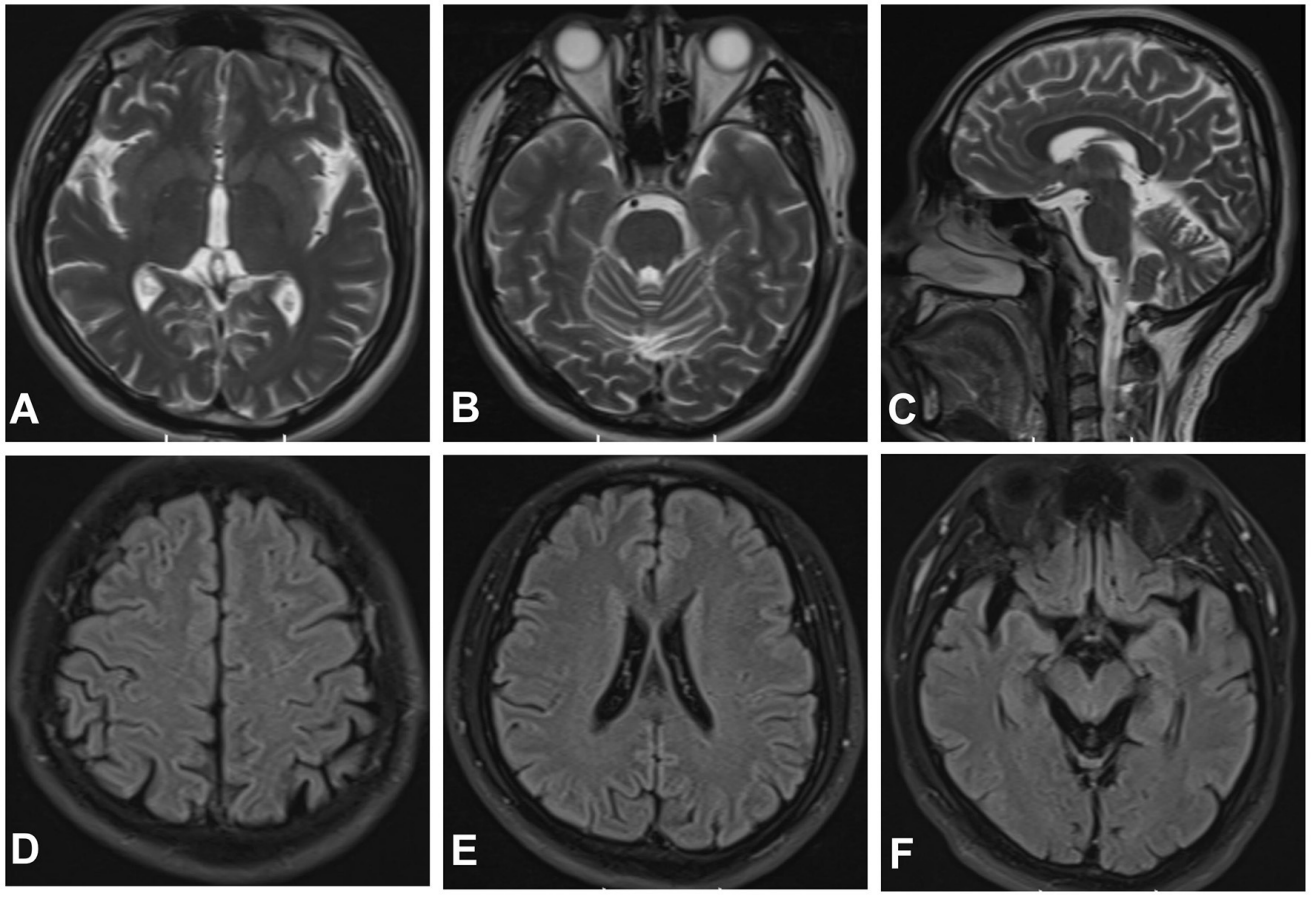
Magnetic resonance imaging (MRI) of the brain. Axial T2-weighted **(A, B)** and sagittal T2-weighted scan **(C)** revealed enlarged sulci in the cerebrum. Fluid-attenuated inversion recovery (FLAIR) sequences **(D–F)** revealed global brain atrophy, ventricular enlargement.

**Figure 3:**
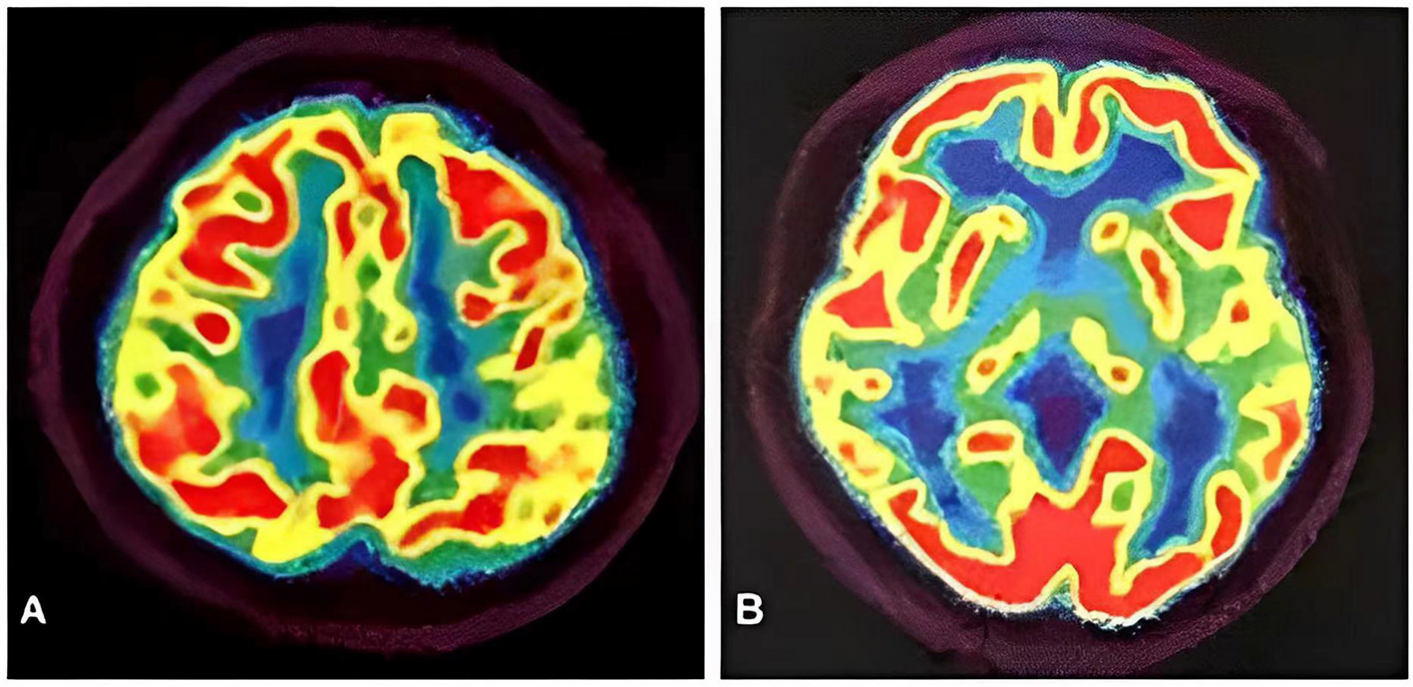
PET-CT images showed the left superior parietal lobe **(A)** and left middle temporal lobe **(B)** had mild to moderate decreased glucose metabolism, with reductions of 10 and 19%, respectively.

**Figure 4:**
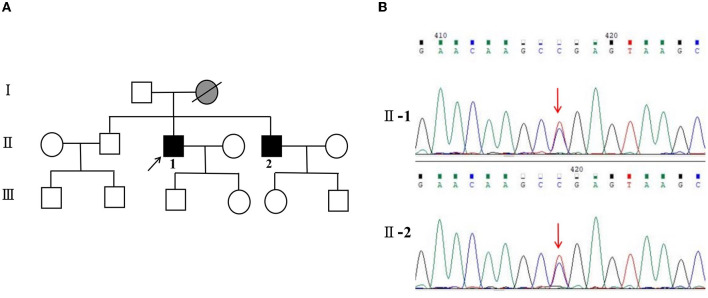
**(A)** Pedigree and PRNP sequences of the proband and his brother. Squares indicate men, circles indicate women, black symbols indicate affected individuals, gray indicates symptoms of presumed GSS, diagonal lines across symbols indicate deceased individuals, and the arrow indicates the proband. 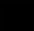 for GSS, and 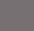 for symptoms of presumed GSS. **(B)** II-1: PRNP sequence of the patient reveals a heterozygous substitution from C to T at position 305 of PRNP cDNA, resulting in an amino acid change from proline to leucine at position 102 (P102L mutation). II-2: PRNP sequence of his little brother confirms the P102L mutation. The arrow indicates the mutation.

Meanwhile, the results of SCA genetic sequencing were found negative. He was referred to our hospital in April 2022. The physical examination revealed mild dysarthria, gait ataxia, bilateral lower extremity weakness, and areflexia but with present Babinski responses bilaterally. The finger-to-nose and rapid alternating movement tests were both abnormal. Orientation, attention, calculation, comprehension, and memory were normal. Laboratory tests and cerebrospinal fluid evaluation were found normal, including the screening for paraneoplastic syndromes-related antibodies and evaluation of 14-3-3 protein levels. Blood and cerebrospinal fluid (CSF) tests were negative for neuromyelitis optica (NMO)-IgG, aquaporin 4 antibodies (AQP4-Ab), and paraneoplastic antibodies. His cognitive function was slightly impaired, and a Mini-Mental State Examination (MMSE) score of 27/30 was obtained during a neuropsychological examination. The interictal electroencephalogram (EEG) showed normal findings (Figure 1). Evoked potential: increase in the binaural threshold. The lower extremity deep sensory path revealed prolonged bilateral P40 latency with amplitude decrease. Brain MRI exhibited T2-weighted and fluid-attenuated inversion recovery (FLAIR) sequences, as well as global brain atrophy, ventricular enlargement and cerebellar atrophy. Diffusion-weighted imaging (DWI) revealed no other abnormalities (Figure 2). PET-CT (18F-fluoro-2-deoxy-d-glucose, FDG) images showed that the left superior parietal lobe and left middle temporal lobe had mild to moderate decreased glucose metabolism, with reductions of 10 and 19%, respectively (Figure 3). We questioned the possible diagnosis of autosomal-recessive cerebellar ataxia (ARCA) before hospitalization, but not exclude a dominant ataxia. Our case was initially diagnosed with SCA. However, the genes responsible for common subtypes of SCA (including SCA1/2/3/6/7/8/12/17, FRDA, and DRPLA) were sequenced for this proband, revealing no pathogenic mutations. The patient was then suspected of having spastic paraplegia; however, areflexia was inexplicable, although later autosomal dominant spastic paraplegia type 4 had a suspected pathogenic site on chromosome 17 (c.1786G>A). The whole-exome sequencing (WES) analysis identified pathogenic heterozygous missense mutations of the *PRNP* gene, c.305C>T (p.Pro102Leu). The Sanger sequencing confirmed that his younger brother inherited the same mutations from his parents (Figure 4). The codon 129 genotype of the patient and his young brother were both P102L-129M/M. His younger brother inherited the same mutations from his parents at the age of 39 years. Up to now, his younger brother still has no symptoms. Then, we diagnosed a case of P102L-associated GSS. We suggested a brain biopsy before making a final diagnosis, but the patient refused. There are currently no approved treatments for GSS. He was treated with buspirone (30 mg/day). The patient's limb weakness worsened rapidly. One year after onset, he often fell due to progressive aggravation of walking instability and decreased muscle strength in his lower limbs. Then, 2 years after onset, he began using a wheelchair and was completely paralyzed in bed most of the time.

## Discussion

We described a case of GSS with unusual clinical and genetic features. Since GSS is an autosomal dominant inherited disease, a single allele mutation can increase the risk of developing the disease. The duration of the disease ranges from 1 to 10 years. GSS has a relatively longer survival duration than other prion diseases. GSS with the P102L mutation is a rare genetic prion disease caused by a pathogenic mutation at codon 102 in the prion protein gene, with diverse clinical variability (7). GSS clinical symptoms include cerebellar ataxia and gait disturbance (72%), cognitive decline (80%), extrapyramidal damage (36%), psychiatric symptoms (21%), and myoclonus (15%) (24, 25). A high positivity rate (83.3%) for the family history was found in the present Chinese case of P102L-associated GSS, with slowly progressive cerebellar ataxia in 90% of patients. In contrast, visual disturbances, dystonia, and myoclonus are uncommon in patients with GSS (18). Ufkes et al. have reported a member of the GSS Indiana Kindred with supranuclear palsy, a less common feature in GSS (26). Li et al. reported five patients from China with progressive ataxia with age at onset ranging from 48 to 52 years (49.5 ± 4.51). All these patients were found to have the p.P102L mutation within PRNP (13). Of course, the vast majority of GSS cases are due to a missense mutation in the PRNP gene although there are a few other reports such as OPRI (27). From 1992 to the present, not much has been reported about Chinese cases of P102L-associated GSS (Tables 1, 2).

**Table 2:** Comparison of basic features of GSS cases with P102L mutation previously reported.

**References**	**Patient no**.	**Origin**	**Sex/age at onset**	**Clinical symptoms**	**Genotype**	**14-3-3**	**EEG**	**Neuroimaging**	**Neuropathology**
Kretzschmar et al. (28)	1–3	Italian	3 cases; 39 y−51 y/N	Dementia, muscular atrophy, cerebellar ataxia	P102L	N	N	N	N
Young et al. (2)	4–5	Canadian	2 cases; 31 y/N, 56 y/N	Mild cognitive impairment; tremor; dysarthria; ataxic gait;	P102L−129M	N	N	Normal	Amyloid plaques, spongiform changes, multi-centric PrP deposits
Barbanti et al. (29)	6–13	Italian	8 cases; 47 y−70 y	Dementia severe ataxia; or ataxia slowly evolving no cognitive impairment	P102L–M/M129	N	Normal or diffuse slow waves	Cortical atrophy	Spongiform changes, multi-centric, uni-centric and “kuru-like” amyloid plaques
Tanaka et al. (4)	14–19	Japanese	6 cases; 1 family, 2 in detail, 34 y/m, 64 y/m	Mental deterioration, speaking and writing difficulty, reduction in verbal fluency, mild ataxia, and wide based gait	P102L–E219K	N	Normal	Atrophy in the cerebral cortex, multiple ischemic lesions	No spongiform changes, no neuronal loss, mild to moderate gliosis, diffuse cortical plaques
Young et al. (30)	20	American	1 case; 33 y/m	Seizures numbness on lower extremities, weakness, dysarthria, swallowing difficulty	P102L−129V	N	N	N	PrP positive amyloid plaque in cortex, hippocampi, caudate, putamen, thalamus. No spongiform changes
Yamada et al. (5)	21–22	Japanese	2 cases; 39 y/f, 35 y/f	Dysarthria, ataxic gait, dysesthesia painful paresthesias, speech disturbances, dysarthria, nystagmus, areflexia in the legs	P102L−129M/M	N	Frequent bursts of theta waves in frontal leads without periodic synchronous discharges	Slight cerebellar atrophy (Case 1)	Spongiform changes in the cerebral and cerebellar cortices, kuru-type plaques, PrP deposits in brain and spina
Majtényi et al. (31)	23–25	Hungarian	3 cases: sisters-56–66 y/f	Visual agnosia, hemiparesis, rigidity, dystonia; paresthesias, dysarthria, dementia, ataxia; behavioral changes	P102L–M129	N	Generalized periodic spike and slow wave activity	Not performed; CT was normal	Spongiform changes, PrP positive uni-centric “kuru” or multi-centric plaques
Bianca et al. (32)	26	Italian	1 case; 41 y/m	Depression, psychosis, dysarthria, ataxia, gait disturbances, limb numbness	P102L–V129	N	Normal	Normal	N
De Michele et al. (33)	27–37	Italian	11 cases; 22 y−71 y	Limb dysesthesias, gait, ataxia, nystagmus, dysmetria, dysarthria, depression, dementia; disorientation, insomnia, apraxia, hyperreflexia, speech disturbance;	P102L	N	Diffuse slowing and spikes in temporal lobes in two cases	Brain and cerebellar atrophy in two cases	Cerebellar slides decreased number of Purkinje cells, uni-centric kuru-type eosinophilic plaques, absence of spongiform changes
Arata et al. (6)	38–48	Japanese	11 cases; 38–70 y (nine families)	Gait disturbance; dysesthesia; hyporeflexa of lower legs; truncal ataxia; leg muscle weakness; dementia and mutism	P102L–M129	Positive in two patients	Normal	High-intensity cerebral cortex, lesions in occipital lobes; others: atrophy	N
Wang et al. (7)	49	Chinese	33 y/F	Dementia and cerebellar ataxia rapidly progressing; language and cognition became progressively more disturbed	P02L	N	Paroxysmal slow waves without periodic synchronous discharges	The upper thoracic segments and mild cerebellar atrophy	Moderate spongiform changes and neuronal loss in the cerebral cortices; proliferation of hypertrophic astrocytes in the cerebral cortices diffuse amyloid plaques in the cerebral cortices; amyloid plaques showed strong immunopositivity by anti-PrP
Giovagnoli et al. (34)	50	Italian	1 case; 31 y/m	Headache, sweating, dysarthria, pyramidal signs, late dementia, mutism and myoclonus	P102L	N	Incomplete periodic synchronous discharges	High intensities in bilateral caudate nuclei, thalami, cerebral cortices	N
Cagnoli et al. (35)	51	Italian	1 case; 52/f	Cerebellar ataxia, frequent falls, dysmetria, hyper-reflexia, Late akinetic mutism	P102L	N	Normal	Normal	N
Chi et al. (8)	52–58	Taiwan	7 cases; 37–53 y	Difficulty to walk, leg weakness, unsteadiness, dysarthria, depression	P102L–M129	N	1 Case: diffuse slow activity; Others: normal	3 Cases: mild cerebellar atrophy, Others: normal	N
Min Jeong Park et al. (9)	59	Korea	1 case; 46 y/f	Slowly progressive ataxia; cognitive decline; dysarthria; severe dementia; dyskinesias	P102L	Positive	Non-specific generalized theta–delta slow waves	Hyperintensities over the entire hemispheric cortices	N
Takazawa et al. (10)	60	Japanese	1 case; 38 y/f	Dysarthria, agraphia, cerebellar ataxia, insomnia; leg hyperreflexia	P102L–M129	Positive	Diffuse theta and delta waves	Vermis atrophy, fronto-parietal cortical high signal	N
Robert Rusina et al. (36)	61	Czech	1 case; 44 y/f	Early personality and behavior changes; paresthesias and ataxia; Memory problems; syoclonus; spasticity; severe dysexecutive impairment	P102– 129M/M	Negative	Generalized triphasic periodic complexes	Caudate and insular hyperintensities	Spongiform dystrophy, prominent amyloid plaques in the cerebellar and cerebral cortex, subcortical gray matter structures, anti-PrP antibodies positivity, amyloid plaques
Miguel A. Riudavets et al. (37)	62–63	Argentine	1family: 2 cases; 50 y/f, 41 y/f	Ataxia; cognitive decline, developed passivity, aphasia, memory loss and agnosia	P102L	N	N	High signal intensity in the basal ganglia	Spongiform changes in cortical layers and basal ganglia deposits of PrP in the Ammon's horn and in the dentate Gyrus; PrP-positive deposits in the amygdala
Yasushi Iwasaki et al. (11)	64	Japanese	54 y/f	Dementia and gait disturbance; bedridden state with myoclonus, akinetic mutism state	P102L	N	Diffuse slowing without periodic sharp-wave complexes	Widespread cerebral cortical hyperintensity	Numerous PrP immunopositive plaques and diffuse synaptic-type PrP deposition were extensively observed, particularly in the cerebral and cerebellar cortices
Chizoba C. Umeh et al. (38)	65	American	56 y/f	Rapidly progressing parkinsonism, dysphasia, dysarthria, and apraxia and dystonia	P102L-129M	N	N	Progressive, global volume loss and hyperintensity in the neocortex and basal ganglia	Neuronal loss, gliosis, spongiform changes, and PrP deposition in the striatum; PrP immunohistochemistry revealed widespread, severe PrP deposition in the thalamus and cerebellar cortex
Long et al. (12)	66	Chinese	47 y/f	Unstable gait and dysarthria; speech slurred; dementia, anxiety, depression, hallucinations or delusions	P102L	N	Normal	Cavum vergae, and mild diffuse brain atrophy; intervertebral herniation in C5/6 and C6/7	N
Li et al. (13)	67–71	Chinese	5 cases: 43 y-55 y	Unsteady walking, dysarthria, dysphagia, changes in personality and irritation, constipation, increased salivation, somnipathy dyssomnia, dementia	P102L	N	Normal	Normal	N
L. Mumoli et al. (39)	72	Italy	32 y/f	Ataxia, cognitive impairment, progressive myoclonus epilepsy	P102L	N	Generalized spike and polyspike waves with a photoparoxysmal response	MRI: brainstem and cerebellar atrophy; PET: severe decrease metabolism in the cerebellum	N
Atsuhiko Sugiyama et al. (14)	73–74	Japanese	2 cases: 55 y/f, 66 y/f	Developed difficulty in using chopsticks, mild speech slurring, subtle dysphagia	P102L	N	N	Atrophy of the cerebellar vermis and brainstem; hyperintensity in the medial portion of both thalami and both pulvinars	N
Jerusa Smid et al. (40)	75–81	Brasil	7 cases; 27–66 y	Dementia; ataxia; paresthesias; myoclonus; epilepsy; parkinsonian syndrome		2 cases Negative	Normal	Cerebellar and cerebral atrophy; frontal atrophy; frontal and parietal cortex hyperintensities	Multicentric plaques in the molecular layer of the cerebellum; multicentric plaque adjacent to granular cells of the dentate fascia of the hippocampus
Wang et al. (15)	82	Chinese	1 case; 49 y/f	Progressive unsteady gait in early stage; progressive dementia; myoclonus; akinetic mutism	P102L	Positive	Dispersedly distributed medium waves together with sharp waves that discharged paroxysmally	Enlarged sulci in cerebellum; high signal intensities in bilateral frontal, parietal, temporal and occipital cortices	N
Michiyoshi Yoshimura et al. (16)	83–87	Japanese	5 cases; 73 y/f, 62 y/f, 61 y/f, 60 y/m, 59 y/m	Ataxia of lower limbs; gait disturbance; dysesthesia in legs; lower limb hyporeflexia	P102L	N	Normal	SPECT and PET: blood flow of anterior cerebellar lobes lower than the posterior cerebellar lobes	N
Areškeviciute A et al. (41)	88	Denmark	1 case 76 y/f	Progressing imbalance, gait disturbance and confusion; cognitive decline; aphasia; double vision; hallucinations	P102L	Positive	Encephalopatic; background slowing pattern and delta activity in frontal area	MRI: abnormalities of right caudate nucleus, slight cortical and central atrophy. PET: generally reduced metabolic.	
Wang et al. (17)	89–100	Chinese	12 cases; 34–67 y	Movement symptoms (gait and walking instability); mental problems (anxiety, dystrophy, irritability); memory decline, dementia	P102L	Positive from 5 cases (45.5%)	2 (25%) of 8 cases exhibited periodic sharp wave complexes	High signal intensities in caudate/putamen (3 cases), DWI ribbon-like signals (3 cases)	N
Zhao et al. (18)	101	Chinese	48 y/m	Unsteady walking; dysarthria; involuntary head tremors; unbearable muscle pain in both lower limbs	P102L	N	Normal	Normal	N
Min Ju Kang et al. (19)	102	Korea	49 y/m	Progressive gait disturbance, slurred speech, clumsiness in both hands; dysarthria and ataxia	P102L	Positive	Normal	Hyperintensities of bilateral cortices; right anterior putamen, right caudate, mild cerebellar atrophy	N
Kazumichi Ota et al. (20)	103–105	Japanese	1family:3 cases; 32 y/m, 53 y/m, 56 y/f	Cognitive function declined; movement symptoms (gait and walking instability), mental problems (behave abnormally); Myoclonus	P102L	Positive	Normal	High signals in occipital and frontal cortices; thalamus and cerebellum mild atrophy	N
Cao et al. (21)	106	Chinese	49 y/m	Unsteady walk with mogilalia; dysdipsia, dysarthria, dizziness, diplopia	P102L	N	N	Cerebral and cerebellar atrophy	N
Yazawa et al. (22)	107	Japanese	56 y/f	Worsening dizziness and walking instability; dysarthria	P102L	N	Periodic focal sharp activity in both temporal areas	Mild atrophy of the cerebellum	N
Hama et al. (42)	108	Japanese	66 y/f	Unsteady gait, cerebellar ataxia; myoclonus of limbs.	P102L	Negative	N	Atrophy of cerebellum, brain stem, cerebellar peduncle, thalamus	N

Genetic testing should be recommended for patients with rapidly progressing paralysis, including gait and balance disorders. Cluster analysis suggests the existence of four clinical phenotypes: typical GSS, GSS with areflexia and paresthesia, pure dementia GSS, and Creutzfeldt-Jakob disease-like GSS (43). The patient had GSS with areflexia. The symptoms at the early stage of the disease should be distinguished from those of hereditary ataxia and spastic paraplegia. Since the patient only presented with ataxia, muscle weakness, and positive family history, hereditary ataxia, such as spinocerebellar ataxia (SCA), should be distinguished.

Non-specific clinical presentation causes delays in diagnosis. Therefore, rare genetic diseases should be paid more attention especially when common causes have been excluded. The patient had no myoclonus, seizures, psychiatric symptoms, parkinsonism, and dementia. We also focused on EEG and 14-3-3 protein in the CSF because typical triphasic complexes and positivity for 14-3-3 protein in patients were useful in confirming the clinical diagnosis of prion disease. In this context, based on the analysis of 12 Chinese patients with P102L-associated GSS disease, Wang et al. found that only one-quarter and less than half of the Chinese patients had periodic sharp wave complexes (PSWC) in EEG and positivity for 14-3-3 protein in the CSF, respectively (17). Coincidental PSWC in EEG and 14-3-3 positivity in the CSF were observed in 50 and 31% of Caucasian GSS patients, respectively (24). Yazawa et al. reported a woman who developed GSS symptoms and was diagnosed with GSS due to the P102L mutation at the age of 58 years. There were no significant EEG findings during the early stage. Bilateral independent periodic discharges (BIPDs) in both temporal areas appeared at the age of 64 years (22), whereas 14-3-3 protein and EEG reports were normal for our patient, making the diagnosis more difficult.

The neuroimaging examination is an essential component in the differential diagnosis. For our patient, the MRI findings did not provide a clear diagnosis. The main imaging features of GSS are cortical atrophy (55.07%), cerebellar atrophy (42.03%), cortical hyperintensities (32.32%), and basal ganglia hyperintensities (21.54%) (43). However, an investigation based on data from the EuroCJD study found FLAIR or DWI hyperintensities in the basal ganglia in 30% of the P102L-associated GSS cases (24). Our patient revealed cortical atrophy and cerebellar atrophy, despite the absence of FLAIR or DWI hyperintensities consistent with GSS. Yoshimura et al. examined five patients from four Japanese families, and predominant abnormalities were found in the occipital and frontal lobes on SPECT and PET analyses, respectively. In SPECT analysis, the blood flow of the anterior cerebellar lobes was lower than that of the posterior cerebellar lobes (44). Hama et al. reported that a Japanese patient with 18F-2-fluorodeoxy-D-glucose (18F-FDG) PET demonstrated hypometabolism of the cerebral cortex, especially in the frontal lobes and thalamus (42). In contrast, we found reduced presynaptic dopamine transporter uptake in the left superior parietal lobe and left medial temporal lobe on PET-CT images. Thus, the significance of MRI findings in P102L-associated GSS needs further evaluation.

Among Japanese P102L-associated GSS cases, 21% presented with early and prominent dementia (45). Another study found that 40% of cases showed cognitive symptoms at the onset (18). However, unlike his mother, our patient had mild cognitive decline. More research in case studies is required to determine whether Chinese P102-associated GSS patients have a higher or lower proportion of cognitive problems. The presence of multicentric prion protein amyloid plaques in neuropathology remains the key feature of GSS that differentiates it from most other genetic prion diseases. There was no diagnosis for 3 years in the present case. Therefore, we do not have the pathological information of the patient. Nonno et al. demonstrated that GSS is a genuine prion disease characterized by both transmissibility and strain variation, expanding our understanding of the heterogeneous clinic-pathological phenotypes of GSS (46).

Our case highlights the clinical heterogeneity of GSS with the most common p.P102L mutation in the family screening. His younger brother showed no symptoms despite carrying the same P102L mutation in the *PRNP* gene. His mother walked unsteadily, eventually unable to walk until her death. Therefore, we inferred that his mother suffered from GSS, although the genetic screening was unavailable. His onset began earlier when he and his family refused to do a brain biopsy. His son and daughter were unaffected but did not consent to *PRNP* gene analysis. Therefore, we do not have full access to the genetic information of the entire family. Penetrance, age of onset, and duration of illness have been systematically characterized across PRNP variants in a global cohort. A genetic counseling session may be triggered by a symptomatic case within the family and may occur either before or after the patient has been tested. Other members of the family, including children need to be able to access clinical services for genetic counseling and testing (47). Several limitations are included in the study. Firstly, we were unable to obtain neuropathological data since the patient did not consent to brain biopsy. Secondly, we have not fully obtained the genetic information of the entire family due to the patient's compliance.

In summary, PRNP sequencing is an indispensable tool for diagnosing GSS due to the complexity of the clinical manifestations of GSS patients. The weakness of the patient's lower limbs developed rapidly, and he arrived at our hospital in a wheelchair. The patient was recently followed up, the strength of his upper limbs was still weak, and he is currently bedridden. However, the patient's younger brother remains asymptomatic.

## Data availability statement

The original contributions presented in the study are included in the article/Supplementary material, further inquiries can be directed to the corresponding authors.

## Ethics statement

The studies involving human participants were reviewed and approved by Ethics Committee of the Affiliated Hospital of the Institute of Neurology of Anhui University of Chinese Medicine. The patients/participants provided their written informed consent to participate in this study. Written informed consent was obtained from the participant/patient(s) for the publication of this case report.

## Author contributions

LC and YX wrote the manuscripts with input from all authors. All authors contributed to data acquisition and analysis. All authors contributed to the article and approved the submitted version.
